# Giant Congenital Melanocytic Nevus of the Buttock

**Published:** 2015-06-17

**Authors:** Andrew A. Marano, Adam M. Feintisch, Ramazi Datiashvili

**Affiliations:** Division of Plastic Surgery, Department of Surgery, Rutgers New Jersey Medical School, Newark

**Keywords:** congenital melanocytic nevus, buttock, serial excision, skin graft, dermal matrix

## DESCRIPTION

A 2-year-old boy with a giant congenital melanocytic nevus (CMN) of the left buttock.

## QUESTIONS

1. **What are congenital melanocytic nevi and how do they present?**
2. **How are congenital melanocytic nevi classified?**
3. **What are the potential risks associated with congenital melanocytic nevi?**
4. **What are the treatment options?**

## DISCUSSION

Congenital melanocytic nevi are abnormal collections of melanocytes that arise in ectopic locations. Melanoblasts begin to develop at 5 weeks' gestation and later migrate from the neural crest to various locations, including the skin, mucosa, eyes, and meninges. Melanocytic nevi arise when dysregulation leads to aberrant growth. Congenital melanocytic nevi are relatively common, occurring at a rate of approximately 1% in the general population. The incidence of giant CMNs is approximately 1 in 20,000, with a female predominance. These lesions typically present as hyperpigmented, flat lesions that change appearance with age. As a child grows, CMNs can undergo color variegation, hypertrichosis, nodular formation, and ulceration. They most commonly occur on the trunk, followed by the extremities and head and neck. There are often smaller satellite nevi dispersed at other locations across the body. Lesions may be associated with pruritus or ulceration. These lesions often have significant impact. The differential diagnosis includes nonmelanocytic nevi such as the epidermal nevus, nevus sebaceous, or nevus flammeus, as well as capillary malformations.^1^

Lesions are typically classified as either congenital or acquired, depending on whether or not the lesion was present at birth. As variability in pigmentation may make diagnosis at birth quite difficult, a third category of tardive melanocytic nevi is used to describe lesions that existed at birth but became apparent within the first 2 years of life.^1^ Congenital melanocytic nevi can be further classified by size: small nevi with diameter of less than 1.5 cm, and medium nevi with diameters between 1.5 and 19.9 cm. They are classified as giant if they exceed 20 cm at their greatest diameter, occupy 2% or more of the total body surface area, or are predicted to reach 20 cm by adulthood.^2^

Giant CMNs may be associated with neurocutaneous melanosis, a rare syndrome in which a giant CMN or multiple smaller CMNs are accompanied by melanocytic deposition in the brain and the spinal cord. There is a bimodal age distribution, with peak incidences in the second to third year of life and second to third decade of life. Risk factors for neurocutaneous melanosis are midline nevi and the presence of more than 20 satellite nevi. Symptoms include hydrocephalus, seizures, developmental delay, and cranial nerve palsy. Prognosis is poor in symptomatic patients, with death usually occurring within 2 to 3 years of diagnosis.^3^ Congenital melanocytic nevi can also undergo malignant conversion, and although the frequency varies widely throughout the literature, most report a 1% to 5% transformation rate.^4^^,^^5^ The lifetime risk for malignant melanoma in a patient with a CMN is approximately 6.3%, or a 17-fold increase compared with the general population. Risk factors for malignant conversion include 3 or more nevi, nevi greater than 20 cm in diameter, and a younger age of onset.^1^ Because malignancy rates increase with age, many surgeons stress the importance of early and complete excision.

The main surgical modalities used to treat CMNs are serial excision, tissue expansion, and skin grafting. Serial excisions are performed when the lesion can be excised in 2 to 3 stages. Approximately 6 months between excisions should be allowed so that tissues have ample time to recover. Serial excision has a favorable complication profile and offers patients time for psychosocial adjustment due to the gradual correction of the nevus. It is, however, limited to lesions that can be excised in about 3 stages. If additional surgical procedures are anticipated, tissue expansion may be preferable. Expansion typically occurs over a course of 3 to 6 months. Potential complications include extrusion, rupture, implant malrotation/malpositioning, flap ischemia, hematoma, and infection, as well as the psychosocial burden among children.^6^ The use of total excision, followed by Integra and split-thickness skin grafting, has been described in the treatment of giant CMNs. Schiestl et al^7^ reported high take rates and excellent functional and cosmetic outcomes using this method on nevi covering up to 12% of the total body surface area. Disadvantages of this technique are its high cost and susceptibility to infection before biointegration of the template.

Our patient underwent primary excision of a giant gluteal CMN and placement of an Integra bilayer matrix dressing. The defect measured 12 × 8 cm. The graft was held in place with the use of a polyurethane sponge dressing for 2 weeks. Avoiding infections in gluteal and perineal areas may be difficult, particularly in children. Hygiene and patient education were of particular importance postoperatively. The patient subsequently underwent split-thickness skin grafting to the area. He was afforded a good cosmetic result, with great color match, texture, and no contour abnormality. Although he developed moderate hypertrophic scarring, he has not had any recurrences 1 year postoperatively. Currently, he undergoes local treatment of hypertrophic scarring.

## Figures and Tables

**Figure 1:**
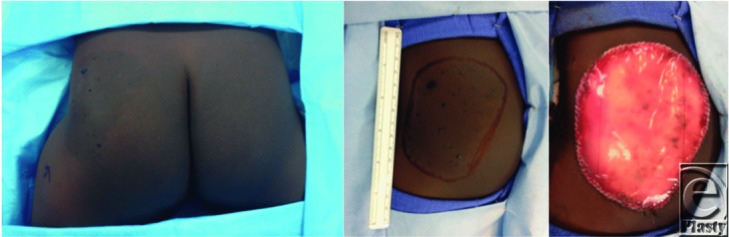
Two-year-old boy with a giant congenital melanocytic nevus of the left buttock. Left, Center, Preoperative views. Right, Immediate view after excision and placement of the Integra bilayer matrix dressing.

**Figure 2:**
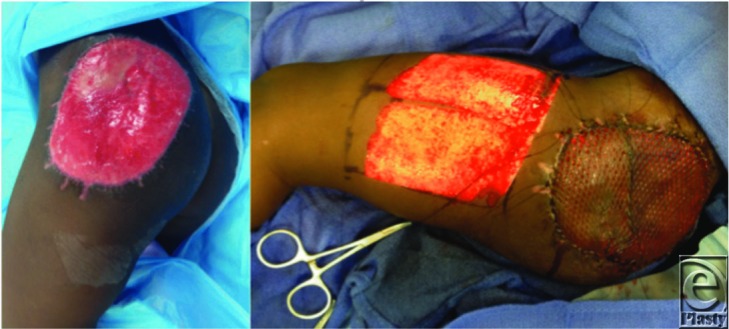
Left, Wound after removal of the silicone layer of the Integra bilayer matrix dressing. Right, Coverage of the wound with a split skin autograft.

**Figure 3:**
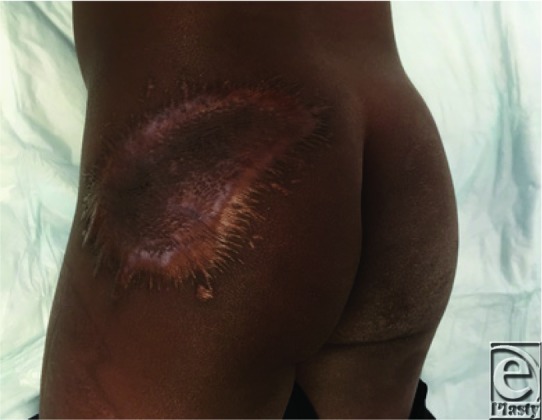
One-year postoperative view. Patient maintains stable coverage; however, he developed some hypertrophic scarring.
